# French Adaptation of the Strengths Use Scale

**DOI:** 10.1177/07342829231205811

**Published:** 2023-10-05

**Authors:** Nicolas Bressoud, Rebecca Shankland, Philippe Dubreuil, Jacques Forest, Karel Belleville, Andrea C. Samson, Philippe Gay

**Affiliations:** 1 129276 Valais University of Teacher Education, St-Maurice, Switzerland; 2 Department of Special Education, University of Fribourg, Fribourg, Switzerland; 3 Laboratoire DIPHE (Développement, Individu, Processus, Handicap, Education), Department of Psychology, Education and Vulnerabilities, 14847 Université Lumière Lyon 2, Lyon, France; 4 Institut Universitaire de France, Paris, France; 5 Department of Human Resources Management, Université du Québec à Trois-Rivières, Trois-Rivières, Canada; 6 Department of Organization and Human Resources, ESG UQAM, Montréal, Québec, Canada; 7 Department of Psychology, 7321 Université de Sherbrooke, Sherbrooke, QC, Canada; 8 Faculty of Psychology, 197131 UniDistance Suisse, Brig, Switzerland; 9 UER EN, 87681 Vaud University of teacher education, Lausanne, Switzerland

**Keywords:** factor analysis < measurement, strengths-based assessment < assessment of interventions/outcomes, educational psychology, test adaptations < culture/crosscultural

## Abstract

**Background:**

Positive psychology focuses on enhancing attitudes and behaviors that support well-being, with a key pillar being the use of psychological strengths for optimal functioning. This is linked to positive outcomes such as increased happiness and life satisfaction.

**Objective:**

This study aimed to evaluate the psychometric validity of the French adaptation of the Strengths Use Scale (SUS), a self-report tool measuring how individuals use their strengths in daily life. The original SUS, developed by Govindji and Linley (2007), has not been thoroughly assessed across languages and cultures.

**Method:**

The French SUS’s psychometric properties were examined using data from six independent French-speaking Canadian samples (*N* = 1397). After removing cases with missing data, exploratory factor analysis (EFA) was conducted on a subsample to establish the optimal factor structure. Confirmatory factor analysis (CFA) was then performed to assess the factor structure’s goodness-of-fit.

**Results:**

Both EFA and CFA supported a unidimensional structure of the scale. The French SUS demonstrated good internal consistency (α = .94). The one-factor model yielded an RMSEA of .122, indicating some model misspecification. However, allowing residuals of some items to covary improved the model fit (RMSEA = .077).

**Conclusion:**

The adapted French SUS exhibits similar properties to the original and presents no new consistency issues. This study contributes to adapting and validating the SUS in French for research and clinical practice. Future research should focus on developing a shorter version by eliminating redundancies and adapting the scale for children to evaluate positive psychology interventions' efficacy in youth.

## Background

Positive psychology focuses on enhancing positive attitudes and behaviors supporting well-being (Seligman & Csikszentmihalyi, 2000). One of its main pillars is the concept of character strengths, which are crucial for human thriving and flourishing (Niemiec, 2020). Strengths are defined as abilities to act, think, or feel in a way that promotes an individual’s best functioning and performance in pursuit of their desired objectives (Linley & Harrington, 2006). These strengths have been associated with several positive outcomes such as higher levels of happiness, lower depression, and higher life satisfaction (Schutte & Malouff, 2019), as well as higher hope and engagement (Madden et al., 2020), job satisfaction, work performance (Miglianico et al., 2020), and academic performance (Lavy, 2020). Although strengths are generally stable over time (Snow, 2019), they can evolve through targeted interventions (Huber et al., 2017; Lavy, 2020; Madden et al., 2020; Schutte & Malouff, 2019).

The ability to recognize one’s talents and understand their significance is referred to as knowledge of strengths. The use of strengths deals with the motivation to apply these skills and the opportunities to implement them in various situations (Wood et al., 2011). Intervention studies suggest that the use of strengths results in long-lasting change (Miglianico et al., 2020; van Zyl & Rothman, 2019), highlighting the importance of assessing strengths use in daily life.

However, the application of this type of intervention in educational or therapeutic settings must be approached with caution. Precise measurements of the effects of the interventions must accompany the educational or therapeutic intervention (Allen et al., 2022).

One popular measure of strengths use is Govindji & Linley (2007) Strengths Use Scale (SUS). This scale, consisting of 14 self-report items, measures opportunities to use strengths and individual behaviors to utilize them. While its concurrent validity has been established in the US population, studies in other countries, such as Germany, call into question its factorial validity (Huber et al., 2017). The scale’s internal consistency has been shown to vary across different cultures and samples (Bu & Duan, 2020; Govindji & Linley, 2007; McTiernan et al., 2020; Vuorinen et al., 2020; Wood et al., 2011), and its temporal stability has been confirmed in a sample of Dutch adults (Van Zyl et al., 2021).

Although the SUS has been translated and used in several languages, including German (Huber et al., 2017) and Chinese (Bu & Duan, 2020), further validation of its psychometric properties is necessary. Additionally, the scale is already being used in French-speaking contexts, particularly in educational and therapeutic settings (e.g., Forest et al., 2012). However, the psychometric properties of the scale have not yet been evaluated in French.

The present study aims to develop positive psychology research practices in French contexts, specifically regarding character strengths. The study’s goal is to evaluate the internal validity of the French version of the SUS. Exploratory and confirmatory factor analyses will be conducted to evaluate the scale’s internal validity.

## Method

For this project, we used the French version (Forest et al., 2012) which was used across six surveys in Canada (*N* = 1532, see Table 1). The six databases were supplied by co-authors of this article. For further details on the nature and context of the collected data, refer to the Appendix.

**Table 1.** Description of sample

Database	Removed	Remaining	Mean age	Gender female (%)
1	17	525	38.28	74.80
2	9	69	39.70	94.10
3	0	142	37.80	80.70
4	2	123	41.41	79.51
5	0	89	24.00	93.40
6	107	449	22.26	56.48
Total	135	1397	33.91	79.83

Participants with NA values on one or more of the 14 items of the SUS were removed from further analyses (see Table 1 for NA distribution through samples and items). We then selected one out of two participants to test, in one half-sample, the scale’s construct validity by exploratory factor analysis and confirmatory factor analysis in two separate samples.

### Participants

The final sample consisted of 1397 participants. In 3 of the 6 databases, the exact ages of the participants were collected. On the 3 other databases, age categories were used. This does not allow us to know with precision the average or median age of participants. We can, however, note that participants are all aged 19 or over, with some categories exceeding 55. To summarize the data, the average of each age category has been used.

### Measures

The SUS is a 14-item scale developed by Govindji & Linley (2007) to determine the extent to which a person uses their strengths actively and with affinity (e.g., “Most of my time is spent doing things that I am good at doing”). Participants respond on a 7-point Likert scale (ranging from 1 = do not agree at all to 7 = very strongly agree).

The French version of the SUS (Forest et al., 2012) was elaborated through the translation and backtranslation method following Vallerand’s transcultural adaptation process (1989). Mean scores of the SUS’s global score range from 1 to 7, with higher scores indicating higher use of one’s own strength.

## Results

### Preliminary Descriptions of Responses and Data Screening (Full Sample, *N* = 1397)

Univariate normality was explored by calculating the skewness and kurtosis of each item. Under normality, data should have a skewness of 0 and a kurtosis of 3. Absolute values for skewness and kurtosis greater than 3 and 20, respectively, are considered to be extreme (Weston & Gore, 2006). In the present sample, the results showed that skewness ranged from -.86 to -.28 and kurtosis from 2.69 to 4.21. Therefore, we assumed there was no indication of a strong deviation from normality and considered the modeling of factorial analyses with the maximum likelihood estimation method (ML) as appropriate.

### Exploratory Factorial Analysis (First Split)

To determine the number of factors to extract from the French SUS correlation matrix, we followed different methods (a parallel analysis, a Velicer’s minimum average partial test, acceleration factor, and optimal coordinates) which all suggested to retain a unique factor solution. However, two eigenvalues were superior to 1 and the first five eigenvalues were superior than .50 (7.99, 1.05, .84, .66, and .60).

We then performed an EFA with one factor that accounted for 53.8% of the total variance. All items loaded strongly (<.63) on the general factor. Finally, the ICC(C,K)—similar to the Cronbach’s alpha coefficient with a confidence interval (McGraw & Wong, 1996)—indicated a good reliability of the scale (α = .94; 95% CI = [.93, .95]).

### Confirmatory Factorial Analysis (Second Split)

We evaluated fit of a unidimensional model with the Mean Square Error of Approximation (RMSEA; Steiger, 1990)) and the Standardized Root Mean Square Residual (SRMR; Bentler, 1995). These two fit indices are recommended because—in comparison to other indices—they could be less sensitive to small misspecifications of factor structure which are very common in the domain of personality research (Beauducel & Wittmann, 2005). As rule of thumb, values of the SRMR indicate good fit if they are between 0 and .05, and values between .05 and .10 an acceptable fit (Schermelleh-Engel et al., 2003); values of RMSEA indicate good fit if they are between 0 and .05, and values between .05 and .08 an acceptable fit (e.g., Fabrigar et al., 1999).

The one-factor model with all 14 items loading directly on to a single factor (Global Strengths Use) yielded a χ^2^(77) = 672, *p* < .001, an RMSEA = .122, 90% CI = (.115, .130), and an SRMR = .045. The RMSEA, above the .08 cut-off, shows some misspecification of the model. Modification indices suggested letting the residuals of several items to covary.

As in previous studies, we let the residuals of different items covary: item 3 with items 2, 9, and 13; item 4 with item 6; item 7 with items 8 and 12; item 9 with items 10 and 13 (and 3); item 10 with item 14; and item 11 with items 10 and 14.

When estimating a second model with all (11) modification indices above 20, results indicated an acceptable fit (χ2(66) = 322, *p* < .001, an RMSEA = .077, 90% CI = (.069, .086), and an SRMR = .031).

## Discussion

Since strengths use is associated with several positive outcomes (e.g., happiness, life satisfaction, and academic outcomes), our study aimed to evaluate the psychometric validity of the French version of the Strengths Use Scale (SUS) for educators, teachers, and researchers.

Factor analysis results revealed that a unidimensional model with all 14 items loading on a single factor had some misspecifications, as indicated by the first RMSEA above the .08 cut-off. Nevertheless, the model fit improved significantly when allowing residuals of several items to covary. Like previous research (Huber et al., 2017), this suggests that the original one-factor model did not fully capture the complexity of strengths use, and certain items may be related in ways not accounted for initially. The French SUS adaptation demonstrated good internal consistency, evidenced by Cronbach’s alpha coefficient. The SUS French version is comparable to the original and presents no unexpected consistency issues.

Limitations of this study include the use of convenience samples, which may affect the generalizability of findings. The samples cannot be generalized to the target population. Despite these limitations, the study contributes to the adaptation and validation of a French SUS for research and clinical practice.

## Conclusion

In educational or therapeutic contexts, positive psychology interventions should be accompanied by valid measures to document their effects, especially regarding character strengths. The SUS appears to be a promising tool for such interventions. This contribution fills an important gap in studying the internal validity of this scale in French contexts.

Future studies could explore developing a shorter version by eliminating redundancies and creating a child-friendly version to assess the effectiveness of positive psychology interventions with young people. Assessing and understanding individuals' ability to apply their strengths in everyday school life can be particularly useful for educators, teachers, and researchers, as it contributes to understanding and developing well-being in schools.

## Appendix

### Data source:

1. Databank CRHA: Dubreuil et al., 2014
2. Revised databank CRE: Dubreuil et al., 2016
3. Belleville et al., 2019
4. Databank CRDICA: Unpublished dataset owned by Philippe Dubreuil.
5. Databank education T1: Goyette & Dubreuil, 2017
6. Databank HUMAN RELATIONS: Forest et al., 2012
